# In Silico Insights into Carbohydrate-Active Enzymes (CAZymes) of *Bacillus subtilis* T7: Lignin and Polysaccharide Degradation Mechanisms

**DOI:** 10.3390/ijms27156610

**Published:** 2026-07-24

**Authors:** Tawaf Ali Shah, Abdullah Sheikh, Hairul Isalm M. Ibrahim, Ashraf Khalifa, Ayesha Ameen

**Affiliations:** 1 College of Agriculture Engineering and Food Science, Shandong University of Technology, Zibo 255000, China; ayeshamin715@gmail.com; 2 Camel Research Center, King Faisal University, P.O. Box 400, Al-Ahsa 31982, Saudi Arabia; asheikh@kfu.edu.sa; 3 Biological Science Department, College of Science, King Faisal University, P.O. Box 400, Al-Ahsa 31982, Saudi Arabia; himohamed@kfu.edu.sa

**Keywords:** CAZymes, *B. subtilis*, molecular docking, molecular dynamics, lytic polysaccharide monooxygenase, biohydrogen, consolidated bioprocessing, lignin degradation

## Abstract

In silico structural characterization of carbohydrate-active enzymes (CAZymes) in *Bacillus subtilis* T7 reveals mechanistic insight into the strain’s capacity for consolidated bioprocessing of untreated lignocellulosic biomass. Homology models for 13 CAZymes were constructed using SWISS-MODEL, with Cu^2+^ and FAD cofactors incorporated into the AA10 lytic polysaccharide monooxygenase and AA3 oxidoreductase models, respectively. Blind molecular docking across the full protein surface identified energetically favorable binding pockets on GH9 endoglucanase and Abhydrolase_1. Among 12 enzyme–ligand pairs screened, Abhydrolase_1 exhibited the highest affinity for xylotetraose (−7.7 kcal/mol) and GH9 showed the strongest preference for cellotetraose (−7.0 kcal/mol). Site-specific docking confirmed six hydrogen bonds with Gly32, Phe33, Thr34, Ser36, Arg179, and His256, supplemented by two carbon–hydrogen bonds with Ile180 and Ser39, anchoring xylotetraose within the Abhydrolase_1 binding cavity, and seven hydrogen bonds stabilizing cellotetraose in the GH9 catalytic groove, with key contacts at Tyr141, Trp145, Asp194, Trp193, Arg254, Tyr255, and Tyr354. One-hundred nanosecond all-atom molecular dynamics simulations (GROMACS 2023.2, CHARMM36 force field, triplicate runs) confirmed overall structural integrity for both proteins: Abhydrolase_1 maintained a compact conformation (Rg = 18.11 ± 0.09 Å; backbone RMSD 2–3 Å), while GH9 was similarly stable (Rg = 30.36 ± 0.33 Å; RMSD 2–5 Å). Ligand dynamics were more variable—xylotetraose remained bound within the Abhydrolase_1 active site for approximately 75 ns before partial displacement, whereas cellotetraose exhibited dynamic association along the GH9 catalytic channel, consistent with processive substrate translocation in endoglucanases. These computational findings line up with the strain’s experimentally observed hydrolytic clearance zones (cellulase 24.5 mm; xylanase 11.6 mm), 63.4% alkali lignin decolorization, transient accumulation of ferulic acid and vanillin, and a hydrogen yield of 1.41 mol H_2_/mol substrate from untreated food waste. Together they give a molecular-level picture of substrate-specific CAZyme recognition in *B. subtilis* T7 and support its potential as a pretreatment-free platform for lignocellulosic biohydrogen production.

## 1. Introduction

Global energy systems remain heavily reliant on fossil fuels, which currently supply over 80% of primary energy and drive both resource depletion and rising greenhouse gas emissions [1]. Hydrogen presents a viable alternative; its combustion yields only water, and it possesses a high gravimetric energy density of 142 MJ kg^−1^ [2]. Concurrently, approximately 1.3 billion tonnes of lignocellulosic organic waste are generated annually from food residues, agricultural crops, and forestry by-products [3,4]. This biomass represents a low-cost, renewable feedstock capable of supporting waste reduction and energy security if converted efficiently into biofuels [5,6,7]. In dark fermentation, Clostridium species typically achieve yields of 1–2 mol H_2_ mol^−1^ hexose. These values remain well below the theoretical maximum of 4 mol H_2_ mol^−1^ hexose achievable via the acetate pathway. Furthermore, most clostridia lack native ligninolytic activity, necessitating upstream pretreatment or syntrophic consortia—steps that add process complexity and limit scalability [8,9].

Lignocellulosic biomass contains cellulose (40–50%), hemicellulose (20–40%), and lignin (10–30%). The polyphenolic lignin matrix shields the polysaccharide components and limits enzymatic access, which creates the main technical barrier in biorefining. Traditional physicochemical pretreatments—such as acid or alkaline hydrolysis, steam explosion, and organosolve methods—break lignin barriers but consume large amounts of energy, require expensive equipment, and often produce fermentation inhibitors such as furfural and phenolic compounds. Biological approaches that operate under mild conditions can preserve carbohydrates, avoid inhibitor formation, and align with sustainable process design [10,11]. Dark fermentation serves as a proven route for biological hydrogen production. *Clostridial* species routinely reach 1–2 mol H_2_/mol hexose, yet these values remain well below the theoretical maximum of 4 mol H_2_/mol hexose through the acetate pathway. Most clostridia lack native ligninolytic activity and therefore need upstream pretreatment or syntrophic consortia, both of which add complexity and reduce scalability. Aerobic lignin-degrading bacteria, including certain *Pseudomonas* and *Rhodococcus* strains, typically mineralize aromatic compounds to CO_2_ rather than directing released sugars toward hydrogen formation [12,13]. In *Bacillus* and related facultative anaerobes, hydrogen evolution during dark fermentation proceeds primarily through two distinct routes. The first, the PFOR–ferredoxin–hydrogenase axis, involves the oxidative decarboxylation of glycolytic pyruvate to acetyl-CoA and CO_2_ by pyruvate:ferredoxin oxidoreductase (PFOR; EC 1.2.7.1). This reaction transfers electrons to ferredoxin, which is subsequently re-oxidized by [FeFe]- or [NiFe]-hydrogenases to release H_2_. While this pathway is canonical in strict anaerobes like Clostridium, it also operates in specific *Bacillus* strains under low-redox conditions. A second route, the formate hydrogen-lyase (FHL) pathway, couples pyruvate formate-lyase (PFL) to a [NiFe]-hydrogenase via formate dehydrogenase. This system dominates in Enterobacteriaceae but is also functional in select *Bacillus* species (e.g., *B. licheniformis*) when glucose is abundant. Theoretical stoichiometry dictates a maximum yield of 4 mol H_2_ mol^−1^ hexose via the acetate branch of the PFOR pathway. However, mixed acetate/butyrate fermentation typically restricts practical yields to 2.0–2.5 mol H_2_ mol^−1^ hexose due to competing NADH-consuming pathways [14,15,16].

Genomically, the *Bacillus* hydrogenase complement is overwhelmingly dominated by [NiFe]-enzymes; bona fide [FeFe]-hydrogenases are rare in the genus. Recent genomic surveys confirm that while most B. subtilis strains lack these genes, the newly isolated strain *B. subtilis* T7 encodes a putative [FeFe]-hydrogenase catalytic subunit. This anomaly provides the structural rationale for the docking and molecular dynamics analyses presented here. *B. subtilis* T7 is a mesophilic strain isolated from anaerobic digester sludge treating mixed municipal food waste and agricultural residues. Taxonomic identification was confirmed by 16S rRNA gene sequencing and Average Nucleotide Identity (ANI > 98.5% relative to *B. subtilis* 168). The draft genome is deposited at NCBI under accession JBSSLY000000000 (BioSample SAMN51985943; BioProject PRJNA1335374). Unlike typical *B. subtilis* isolates, strain T7 exhibits simultaneous lignin modification and hydrogen production from untreated substrates in monoculture. It encodes a broad suite of oxidative and hydrolytic CAZymes alongside the functional [FeFe]-hydrogenase, positioning it as a unique single-organism platform for consolidated bioprocessing (CBP). *Bacillus* species are facultative anaerobes characterized by rapid growth and efficient secretion of extracellular enzymes. Several species, including *B. subtilis*, *B. licheniformis*, and *B. amyloliquefaciens*, hold Generally Recognized as Safe (GRAS) status from the U.S. FDA [17]. However, this designation is strictly species- and strain-specific; it does not extend to pathogenic members of the genus such as *B. cereus* or *B. anthracis*, necessitating rigorous safety validation for any new industrial isolate [18].

**Figure 1:**
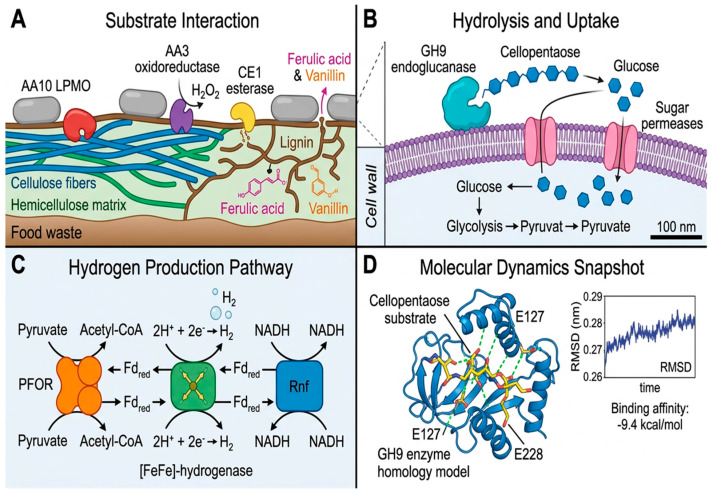
Integrated lignocellulose deconstruction and hydrogen production by *B. subtilis* T7 in consolidated bioprocessing. (**A**) Schematic representation of *B. subtilis* T7 adhering to untreated food waste, secreting oxidative CAZymes (AA10, AA3, CE1) that disrupt the lignin matrix (brown) and hydrolyze cellulose (blue) and hemicellulose (green). Released aromatic monomers (ferulic acid, vanillin) and cello-oligosaccharides are shown. (**B**) Docking of cellotetraose with GH9 endoglucanase, showing the seven conventional hydrogen-bond interactions with residues Tyr141, Trp145, Asp194, Arg254, Tyr255 and Tyr354, together with a π-σ interaction involving Trp193. The 3D surface representation highlights the accommodation of cellotetraose within the substrate-binding groove and a favorable binding orientation supported by extensive polar interactions, suggesting efficient substrate recognition and catalytic potential of the enzyme. (**C**) [FeFe]-hydrogenase catalyzes proton reduction using electrons from Fd_red_, yielding molecular hydrogen (H_2_). Enzymes are labeled with EC numbers: PFOR (1.2.7.1), [FeFe]-hydrogenase (1.12.7.2), Rnf. (**D**) Molecular dynamics snapshot of the GH9 endoglucanase–cellopentaose complex after 100 ns simulation. GH9 (blue ribbon) stably binds cellopentaose (yellow sticks) with persistent hydrogen bonds (green dashes) to catalytic residues E127 and E228. Binding affinity: −9.4 kcal/mol; RMSD: 0.26–0.29 nm.

These traits make them attractive for industrial bioprocessing. Selected *Bacillus* strains produce hydrogen at 1.0–1.8 mol H_2_/mol hexose from pretreated substrates or in co-cultures. Although several *Bacillus* strains exhibit partial lignocellulolytic or hydrogenogenic activities, few combine robust lignin modification with direct fermentative hydrogen evolution from untreated biomass in axenic culture. Earlier studies on *B. subtilis* have largely examined isolated traits, such as cellulolytic activity or hydrogen production from simple sugars, without demonstrating integrated CBP performance. Earlier studies on *B. subtilis* examined isolated functions, such as lignin peroxidase activity in tobacco processing or hydrogen production from simple sugars and fruit waste, but did not integrate lignin depolymerization with fermentative hydrogen evolution [19]. Strains such as *B. subtilis* 1AJ3 display cellulolytic activity on switchgrass, and certain rumen-derived isolates improve rice straw saccharification, yet none couple these traits with hydrogen production in a single organism. Computational work in related studies has often relied on transcriptomics or basic enzyme kinetics and rarely combined in vivo phenotypes with detailed molecular docking and molecular dynamics simulations to clarify substrate binding mechanisms. Moreover, few *B. subtilis* strains possess both oxidative carbohydrate-active enzymes (CAZymes), including AA10 lytic polysaccharide monooxygenases (LPMOs), and functional [FeFe]-hydrogenases (Figure 1). This combination is essential for consolidated bioprocessing (CBP), in which hydrolysis, saccharification, and fermentation occur inside one microorganism. The present study addresses this gap by providing an in silico structural characterization of key carbohydrate-active enzymes CAZymes encoded in the genome of *B. subtilis* T7, a strain isolated from anaerobic digester sludge. The analysis focuses on homology modeling, blind molecular docking of relevant ligands, and 100 ns molecular dynamics simulations of selected enzyme–substrate complexes. These computations aim to supply atomic-level explanations for the strain’s reported experimental performance, including 63.4% decolorization of alkali lignin with transient release of ferulic acid and vanillin, robust hydrolytic clearance zones on polysaccharide plates, and hydrogen yields up to 1.41 mol H_2_/mol substrate from untreated food waste [20].

The work tests the hypothesis that stable binding interactions between GH9 endoglucanase and cellulose oligomers, supported by oxidative activity from AA10 and AA3 enzymes, underpin the observed simultaneous lignin modification and polysaccharide-to-hydrogen conversion without pretreatment or syntrophic partners. Several precursor studies illustrate both progress and remaining limitations. *B. subtilis* 1AJ3 degrades switchgrass under thermochemical pretreatment conditions and expresses a GH16 β-glycoside hydrolase whose docking energy toward a model substrate reached −5.72 kcal/mol. The strain achieved modest acid-insoluble lignin reduction (13.91%) but required pretreatment and did not produce hydrogen. Certain *Bacillus* species, including *B. coagulans*, *B. thuringiensis*, and *B. amyloliquefaciens*, exhibit direct hydrogenogenic capacity during dark fermentation, producing biohydrogen from agricultural wastes and lignocellulosic substrates without requiring pretreatment. Strains such as *B. coagulans* MO11 and IIT-BT S1 achieve hydrogen yields of up to 1.63–2.28 mol H_2_/mol hexose, utilizing substrates like molasses, glucose, and untreated leaf biomass. These facultative anaerobes secrete hydrolytic enzymes and function effectively under mesophilic conditions, offering robustness and metabolic versatility. In contrast, other *Bacillus* strains involved in lignin degradation or saccharification lack hydrogen production, highlighting functional diversity within the genus [20].

This study tests the hypothesis that stable enzyme–substrate interactions, particularly involving the GH9 endoglucanase, together with oxidative activity from AA10 and AA3 family members, underpin the strain’s observed ability to modify lignin and convert polysaccharides to hydrogen without conventional pretreatment.

## 2. Results

### 2.1. Ligninolytic and Hydrolytic Activities of B. subtilis T7

*B. subtilis* T7 exhibited lignin-modifying activity in both solid and liquid culture systems. On minimal salts agar plates supplemented with alkali lignin or Azure B, distinct decolorization zones formed around colonies within 96 h at 37 °C, indicating secretion of extracellular enzymes capable of degrading complex aromatic polymers. In liquid cultures over 7 days, the strain achieved 63.4 ± 3.2% decolorization of alkali lignin (measured at 280 nm) and 56.5 ± 2.9% decolorization of Azure B (measured at 663 nm). Bacterial growth was unaffected, reaching an optical density of 1.45 ± 0.12 at 620 nm by day 7 (Supplementary Figure S1). High-performance liquid chromatography (HPLC) analysis of culture supernatants showed transient accumulation of aromatic monomers. Ferulic acid reached a peak concentration of 0.85 ± 0.07 mg/L on day 3, coinciding with a maximum vanillin concentration of 0.67 ± 0.05 mg/L. p-Coumaric acid peaked at 0.32 ± 0.04 mg/L and was largely depleted by day 6, consistent with subsequent utilization.

Solid-medium plate assays demonstrated robust hydrolytic activity. On Congo Red-stained carboxymethyl cellulose (CMC) agar, T7 produced clearance zones of 24.5 ± 1.2 mm, indicating endoglucanase activity. Clearance zones of 11.6 ± 0.8 mm and 14.2 ± 1.0 mm were observed on xylan and starch–iodine plates, respectively, confirming xylanase and amylase activity. Proteolytic activity produced zones of 23.3 ± 1.5 mm on skim milk agar (Supplementary Figure S3). These phenotypic data are consistent with the genomic presence of oxidative CAZymes (AA10, AA3) and multiple glycoside hydrolases, as previously reported [20].

### 2.2. Biohydrogen Production from Various Substrates

In batch dark fermentation experiments, *B. subtilis* T7 produced hydrogen directly from various carbon sources in pure culture without syntrophic partners. The highest volumetric yield was obtained with xylose (274 ± 15 mL H_2_/g VS, equivalent to 0.92 ± 0.05 mol H_2_/mol substrate). Modified Gompertz modeling gave a maximum production rate (R_max_) of 45.2 ± 3.1 mL/h and a lag phase of 12.4 ± 1.2 h. Untreated food waste yielded the highest molar hydrogen output of 1.41 ± 0.08 mol H_2_/mol substrate (210 ± 14 mL H_2_/g VS). Yields from glucose, starch, and carboxymethyl cellulose- CMC ranged from 1.02 to 1.25 mol H_2_/mol substrate. Acetate and butyrate were the dominant volatile fatty acids, and no methane was detected (Table 1). End-of-run volatile fatty acid (VFA) profiles confirm a mixed acetate/butyrate fermentation pathway across all substrate conditions. Acetate and butyrate were the dominant metabolites, with concentrations ranging from 1.3–2.6 g/L and 0.56–1.6 g/L, respectively (Table 1). Propionate, an electron sink often associated with reduced hydrogen yields, remained below the HPLC detection limit (<0.05 g/L) in every reactor. The molar acetate-to-butyrate (A:B) ratio varied by substrate, ranging from 1.31 (food waste) to 2.32 (CMC). While a higher A:B ratio typically favors greater hydrogen production per mole of substrate due to the stoichiometry of the acetate pathway (4 mol H_2_/mol hexose) versus the butyrate pathway (2 mol H_2_/mol hexose), the complex composition of food waste likely influences this trend, yielding the highest molar hydrogen (1.41 mol/mol) despite a lower A:B ratio.

### 2.3. Molecular Docking Analysis of Key CAZymes

Blind docking against the entire solvent-exposed surface of GH9 endoglucanase and Abhydrolase_1 identified multiple energetically favorable binding pockets for carbohydrate ligands without imposing any prior constraint on binding location. The most productive docking conformations consistently clustered within surface-accessible groove regions, topologically consistent with the substrate-binding channels of processive polysaccharide-degrading enzymes. For GH9, dominant poses converged within a large catalytic cleft traversing the enzyme surface—a hallmark geometry for endoglucanases accommodating extended polysaccharide chains. Abhydrolase_1 similarly orientated its best-scoring poses within a polar, well-defined surface pocket lined with hydrogen-bond donors and acceptors. In both cases, the binding sites identified by blind docking agreed closely with the top-ranked pockets predicted by PrankWeb, validating the unbiased approach and providing the structural rationale for subsequent site-specific analyses [21].

The carbohydrate ligands consistently adopted extended conformations along these groove regions, maximizing contact surface with flanking residues—a binding mode structurally analogous to the substrate-engagement geometry of characterized polysaccharide hydrolases. Across all 12 enzyme–ligand combinations tested, Abhydrolase_1 exhibited the highest binding affinity for xylotetraose (−7.7 kcal/mol), while GH9 showed the strongest preference for cellotetraose (−7.0 kcal/mol), suggesting substrate specificity toward hemicellulose- and cellulose-derived oligosaccharides, respectively (Table 2).

Cellopentaose was retained as an additional ligand only for the inter-strain comparison of T7 GH9 with published *Bacillus* GH9 endoglucanases (Supplementary Table S2); the 12 enzyme-ligand combinations described in this section and the molecular dynamics runs that follow were carried out with cellotetraose. Template sequence identities are taken from Supplementary Table S1 and refer to the SWISS-MODEL template chosen for each enzyme. All values are well above the 30% threshold typically considered the minimum for reliable homology modeling, supporting the use of these models for the docking and dynamics calculations reported here.

### 2.4. Site-Specific Molecular Docking of Xylotetraose with Abhydrolase_1

**Figure 2:**
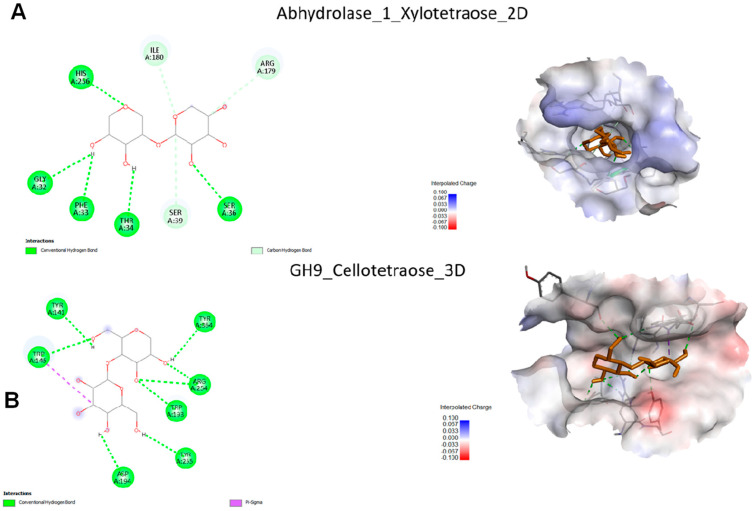
Molecular docking interaction analysis of carbohydrate ligands with glycoside hydrolase enzymes. The left panels show the 2D interaction maps, while the right panels present the corresponding 3D binding conformations within the active-site cavities. (**A**) Docking of xylotetraose with Abhydrolase_1, illustrating conventional hydrogen-bond interactions involving residues Gly32, Phe33, Thr34, Ser36, and His256, which contribute to ligand stabilization within the binding pocket. (**B**) Docking of cellotetraose with GH9 endoglucanase, showing multiple hydrogen-bond interactions with residues Tyr141, Trp145, Asp194, Trp193, Tyr215, Arg254, and Tyr354, along with a π–sigma interaction involving Trp145. The 3D surface representations highlight the accommodation of the ligands within the substrate-binding grooves and demonstrate favorable binding orientations supported by extensive polar interactions, suggesting efficient substrate recognition and catalytic potential of the enzymes.

**Figure 3:**
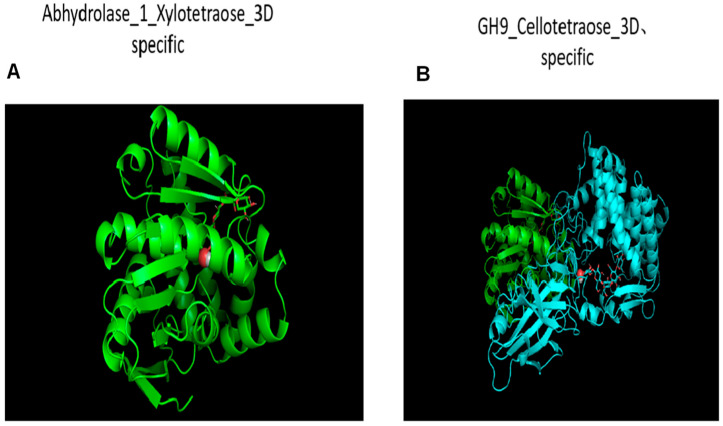
Three-dimensional visualization of protein–ligand docking complexes showing substrate binding within the catalytic pockets of selected enzymes. (**A**) Docked complex of Abhydrolase_1 with xylotetraose, represented as a ribbon model (green) with the ligand shown in stick representation at the active site. The ligand occupies a central binding cavity and is stabilized by interactions with catalytic and substrate-recognition residues, indicating favorable substrate accommodation. (**B**) Docked complex of GH9 endoglucanase with cellotetraose, displayed as a dimeric ribbon structure (cyan and green chains) with the ligand bound along the substrate-binding groove. The binding orientation of cellotetraose suggests efficient recognition of cellulose-derived oligosaccharides and supports the catalytic role of GH9 in cellulose hydrolysis. These structural models demonstrate the accessibility of the active sites and the compatibility of the ligands with their respective enzyme binding pockets.

Site-specific docking of xylotetraose to the top-ranked PrankWeb-predicted cavity of Abhydrolase_1 revealed favorable accommodation of the ligand within a well-defined binding groove (Figure 2 and Figure 3, Table 3). Two-dimensional interaction analysis identified six conventional hydrogen bonds formed with residues Gly32, Phe33, Thr34, Ser36, and His256, constituting the primary stabilizing forces within the complex. Additional carbon–hydrogen bond contacts with Ser39, Arg179, and Ile180 provided supplementary anchoring of xylotetraose within the catalytic pocket. Three-dimensional visualization confirmed that the ligand occupied a central binding cavity with extensive surface complementarity to the enzyme, adopting an orientation consistent with productive substrate engagement. The binding affinity of −7.7 kcal/mol—the highest observed across all enzyme–ligand pairs screened—reflects strong geometric and electronic compatibility between xylotetraose and the Abhydrolase_1 active site.

### 2.5. Site-Specific Molecular Docking of Cellotetraose with GH9

Site-specific docking of cellotetraose against the GH9 active site demonstrated favorable insertion of the tetrasaccharide into the enzyme’s extended catalytic groove. The interaction profile comprised seven conventional hydrogen bonds, with key contacts at Tyr141, Trp145, Tyr255, Tyr354, Arg254, and Asp194. An additional π–sigma interaction with Trp193 contributed further non-covalent stabilization. Three-dimensional modeling showed cellotetraose adopting an extended conformation spanning the full substrate-binding channel, simultaneously engaging both catalytic and substrate-recognition residues. This binding geometry is characteristic of endoglucanases that accommodate multiple glucosyl units and cleave internal glycosidic bonds, and is consistent with the proposed cellulolytic role of GH9 in *B. subtilis* T7. The binding affinity of −7.0 kcal/mol, together with the breadth of polar contacts identified, supports efficient recognition of cellulose-derived oligosaccharides by this enzyme. The primary structures of the two enzymes selected for molecular dynamics simulation using AlphaFold (GH9 endoglucanase and Abhydrolase_1), with the key interacting residues shown in Table 3, are provided as Supplementary Figures S4 and S5.

### 2.6. Molecular Dynamics (MD) Simulation Analysis of Abhydrolase_1–Xylotetraose Complex

**Figure 4:**
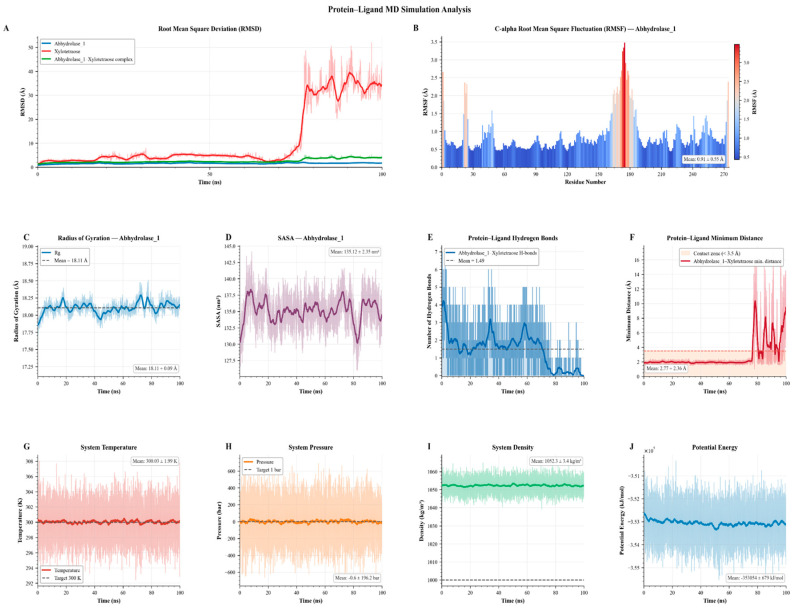
Molecular dynamics simulation of the Abhydrolase_1–Xylotetraose complex. RMSD (**A**), RMSF (**B**), radius of gyration (**C**), SASA (**D**), hydrogen bond occupancy (**E**), minimum protein–ligand distance (**F**), temperature (**G**), pressure (**H**), density (**I**), and potential energy (**J**) were monitored during a 100 ns simulation to evaluate structural stability, conformational flexibility, and binding dynamics of the complex.

The stability and binding dynamics of the Abhydrolase_1–xylotetraose complex were examined over a 100 ns all-atom molecular dynamics trajectory, monitoring RMSD, RMSF, radius of gyration (Rg), solvent-accessible surface area (SASA), hydrogen-bond occupancy, minimum protein–ligand distance, and thermodynamic parameters (Figure 4).

#### 2.6.1. Root Mean Square Deviation (RMSD)

The backbone RMSD of free Abhydrolase_1 stabilized rapidly and fluctuated within 2–3 Å throughout the simulation, indicating an inherently rigid protein scaffold (Figure 4A). Upon ligand binding, the complex exhibited modestly elevated RMSD values of approximately 3–5 Å, reflecting local structural rearrangements associated with substrate accommodation rather than any global disruption of fold. In contrast, xylotetraose underwent progressive displacement after approximately 75 ns, with ligand RMSD rising above 30 Å, signaling substantial migration from the initial docked orientation during the terminal phase of the trajectory.

#### 2.6.2. Root Mean Square Fluctuation (RMSF)

Per-residue RMSF analysis yielded an average fluctuation of 0.91 ± 0.55 Å across the protein backbone, confirming overall structural rigidity (Figure 4B). The large majority of residues remained below 1.5 Å in mobility. A localized region of elevated flexibility was identified around residues 170–180, where RMSF values approached 3.5 Å, suggesting a loop or hinge element that may contribute to dynamic substrate recognition during catalysis.

#### 2.6.3. Radius of Gyration (Rg)

The radius of gyration remained consistent throughout the simulation at an average of 18.11 ± 0.09 Å (Figure 4C), confirming that Abhydrolase_1 retained its compact tertiary fold without appreciable expansion or partial unfolding over the full 100 ns trajectory.

#### 2.6.4. Solvent-Accessible Surface Area (SASA)

The mean SASA was 135.12 ± 2.35 nm^2^, with only minor fluctuations along the trajectory (Figure 4D). The absence of any pronounced directional trend in surface exposure indicates that the protein maintained structural integrity without significant domain rearrangements during the simulation.

#### 2.6.5. Protein–Ligand Hydrogen Bonds

Hydrogen-bond analysis revealed persistent interactions during the initial 75 ns, with an average occupancy of 1.49 bonds and transient peaks of up to 6–7 simultaneously (Figure 4E). The progressive decline in hydrogen-bond occupancy beyond approximately 75 ns coincided directly with the marked rise in ligand RMSD, consistent with partial detachment of xylotetraose from its binding site during the terminal phase of the trajectory.

#### 2.6.6. Protein–Ligand Minimum Distance

Minimum-distance analysis confirmed that xylotetraose remained in close proximity to the binding pocket during the first 70–75 ns, with inter-atomic distances not exceeding 3.5 Å (Figure 4F). Beyond this point, the protein–ligand distance increased substantially to 10–16 Å, indicating partial dissociation or active-site migration of the ligand. The time-averaged minimum distance of 2.77 ± 2.36 Å reflects the predominantly bound state over the majority of the simulation period.

#### 2.6.7. Thermodynamic Stability

System equilibration was confirmed by thermodynamic monitoring: temperature was maintained at 300.03 ± 1.99 K (Figure 4G); pressure fluctuated around the target with a mean of −0.6 ± 196.2 bar (Figure 4H), typical of NPT ensemble simulations; density remained stable at 1052.3 ± 3.4 kg m^−3^ (Figure 4I); and potential energy stabilized at approximately −3.53 × 10^5^ ± 679 kJ mol^−1^ (Figure 4J), consistent with a fully equilibrated system.

Abhydrolase_1 maintained excellent structural stability across the entire 100 ns trajectory, as evidenced by the invariant backbone RMSD, stable radius of gyration, and well-equilibrated thermodynamic parameters. Xylotetraose remained associated with the active site throughout most of the simulation but underwent partial displacement after approximately 75 ns, accompanied by a decline in hydrogen-bond occupancy and a marked increase in protein–ligand distance. This behavior is consistent with a moderate-affinity interaction in which the flexible loop region around residues 170–180 plays a functionally important role in dynamic substrate recognition and binding. The partial dissociation observed in the later stages of the trajectory may reflect a catalytically relevant event—such as product release following glycosidic bond cleavage—rather than an inherent instability of the complex.

### 2.7. Molecular Dynamics (MD) Simulation Analysis of GH9–Cellotetraose Complex

**Figure 5:**
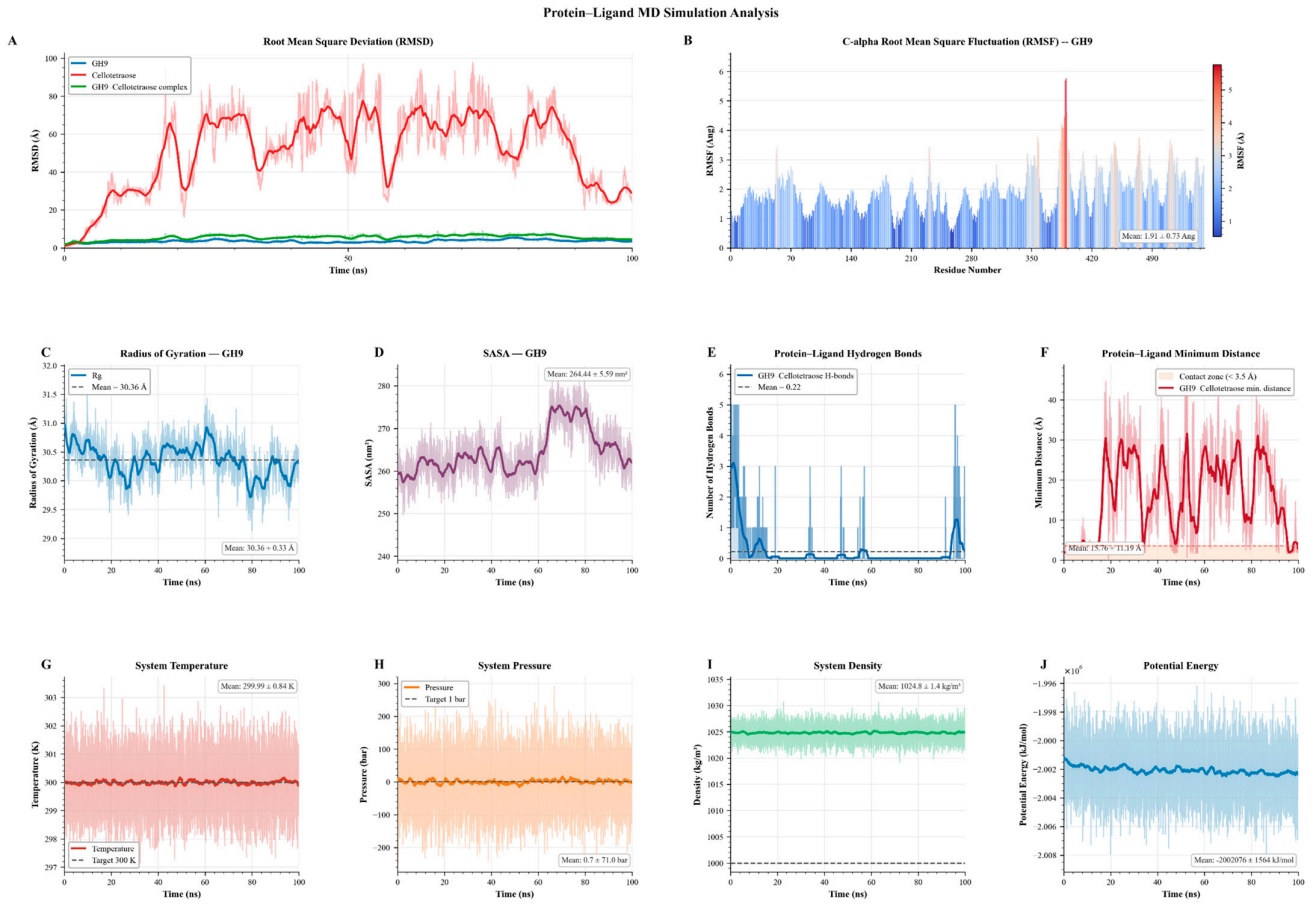
Molecular dynamics simulation analysis of the GH9–cellotetraose complex over 100 ns showing (**A**) RMSD, (**B**) Cα-RMSF, (**C**) radius of gyration (Rg), (**D**) solvent-accessible surface area (SASA), (**E**) protein–ligand hydrogen bonds, (**F**) minimum distance, (**G**) temperature, (**H**) pressure, (**I**) density, and (**J**) potential energy profiles, demonstrating the structural and thermodynamic stability of the complex throughout the simulation.

The GH9–cellotetraose complex was analyzed over a 100 ns all-atom molecular dynamics trajectory using the same suite of structural and thermodynamic metrics (Figure 5).

#### 2.7.1. Root Mean Square Deviation (RMSD)

The GH9 backbone RMSD was stable throughout the 100 ns simulation, fluctuating within 2–5 Å (Figure 5A), consistent with a structurally rigid globular enzyme. The GH9–cellotetraose complex maintained a similar RMSD range of 3–6 Å, indicating that the overall protein architecture was not substantially perturbed by substrate binding. Free cellotetraose in solution, used as a conformational reference, displayed large RMSD fluctuations of 70–90 Å, illustrating the degree to which the GH9 binding channel restricts ligand conformational freedom in the complex.

#### 2.7.2. Residue Flexibility (RMSF)

Per-residue RMSF analysis yielded an average value of 1.91 ± 0.73 Å across all Cα atoms (Figure 5B). Most residues remained below 3 Å in fluctuation; only a loop region around residues 380–400 showed elevated mobility, reaching approximately 5.8 Å, likely corresponding to a flexible surface loop peripheral to the catalytic channel.

#### 2.7.3. Compactness of the Protein (Radius of Gyration)

The radius of gyration averaged 30.36 ± 0.33 Å throughout the simulation (Figure 5C), confirming that GH9 maintained its compact tertiary fold without significant structural expansion or domain separation during the entire 100 ns trajectory.

#### 2.7.4. Solvent Accessibility (SASA)

Mean SASA was 264.44 ± 5.59 nm^2^ (Figure 5D). A transient increase in solvent-exposed surface area was observed between 60 and 80 ns, followed by a return toward baseline, indicating a brief and reversible conformational fluctuation that did not compromise overall structural integrity.

#### 2.7.5. Protein–Ligand Hydrogen Bonds

Hydrogen-bond analysis revealed an average occupancy of 0.22 bonds between GH9 and cellotetraose, with intermittent peaks of up to five simultaneous hydrogen bonds (Figure 5E). This low time-averaged value, punctuated by transient high-occupancy events, is characteristic of a highly dynamic binding interaction in which the oligosaccharide samples multiple positions along the catalytic channel rather than residing in a single fixed conformation.

#### 2.7.6. Protein–Ligand Distance

The average minimum protein–ligand distance was 15.76 ± 11.19 Å, with values ranging from approximately 2 Å to 40 Å during the trajectory (Figure 5F). This broad variation reflects multiple association and dissociation events throughout the simulation, consistent with dynamic substrate sampling across the GH9 surface.

#### 2.7.7. Thermodynamic Stability

The system maintained excellent thermal equilibration at 299.99 ± 0.84 K, close to the set-point temperature of 300 K (Figure 5G). Pressure fluctuated at approximately 0.7 ± 71.0 bar within the expected range for an NPT ensemble (Figure 5H). System density remained stable at 1024.8 ± 1.4 kg m^−3^ (Figure 5I), and the potential energy distribution averaged approximately −2.002 × 10^6^ ± 1564 kJ mol^−1^ (Figure 5J), confirming a well-equilibrated simulation box throughout the production phase.

GH9 itself was structurally stable and compact throughout the entire 100 ns simulation, as demonstrated by the low RMSD, stable radius of gyration, and invariant thermodynamic parameters. Cellotetraose, in contrast, exhibited highly dynamic behavior—brief high-affinity binding events interspersed with periods of ligand displacement—yielding a low mean hydrogen-bond occupancy and substantial variation in protein–ligand distance. Rather than indicating an inherently weak interaction, this pattern is consistent with the processes sliding mechanism proposed for endoglucanases, in which the substrate translocate stepwise along an extended catalytic channel to present successive glycosidic bonds for cleavage. The elevated flexibility observed near residues 380–400 may facilitate this translocation by providing a conformationally mobile surface that accommodates repositioning of the oligosaccharide chain.

## 3. Discussion

Taken together, the molecular docking and molecular dynamics results provide a coherent atomic-level narrative for the lignocellulolytic potential of *B. subtilis* T7. The blind docking surveys confirmed that both GH9 and Abhydrolase_1 possess extensive surface-accessible binding landscapes for oligosaccharide substrates, with favorable poses consistently converging in groove-like cavities topologically concordant with the PrankWeb-predicted active sites. This agreement between two independent computational methods strengthens confidence in the identified binding regions and validates the unbiased blind-docking approach as an effective starting point for characterizing CAZyme–substrate interactions in poorly annotated enzymes.

The substrate preferences that emerged from the docking data are chemically coherent. Abhydrolase_1, the highest-affinity binder in the screen (−7.7 kcal/mol for xylotetraose), recognizes a pentose-backbone ligand through an array of six hydrogen bonds with polar residues including Gly32, Phe33, Thr34, Ser36, His256, Ser39, Arg179, and Ile180. The pocket geometry and residue composition strongly suggest activity on hemicellulosic substrates, likely xylan-derived oligomers released during the early stages of lignocellulose deconstruction. GH9, by contrast, showed highest affinity for cellotetraose (−7.0 kcal/mol) and engaged the ligand through seven hydrogen bonds spanning both catalytic residues (Asp194) and substrate-recognition elements (Tyr141, Trp145, Tyr255, Arg254, Tyr354), together with a stabilizing π–sigma interaction at Trp145. This interaction profile—involving multiple aromatic stacking and polar contacts along an extended groove—is characteristic of family 9 endoglucanases known to act on internal glucosidic bonds in amorphous and crystalline cellulose. The observed binding energy of −7.0 kcal/mol substantially exceeds the −5.72 kcal/mol reported for a GH16 enzyme from *B. subtilis* 1AJ3 docked against a model glucoside, suggesting tighter substrate engagement in T7 GH9 despite both enzymes belonging to the broader GH [22].

The molecular dynamics results add an important dynamic dimension to these docking snapshots. For the Abhydrolase_1–xylotetraose system, the protein structure was remarkably stable across the full 100 ns trajectory, with backbone root mean square deviation RMSD confined to 2–3 Å and radius of gyration Rg invariant at 18.11 ± 0.09 Å—clear indicators of a well-folded, globally rigid protein. The flexible loop around residues 170–180, however, is a structural feature of functional consequence: its elevated RMSF (approaching 3.5 Å) likely facilitates induced-fit accommodation of xylotetraose, and its mobility may also mediate product release. The progressive displacement of xylotetraose after approximately 75 ns—marked by rising ligand root mean square deviation RMSD above 30 Å, diminishing hydrogen-bond occupancy from a peak of 6–7 to near zero, and protein–ligand distances expanding to 10–16 Å—could reflect either the transient character of carbohydrate–enzyme interactions in general or a catalytically productive dissociation event following cleavage of the glycosidic linkage. Either interpretation is mechanistically plausible, and distinguishing between them would require complementary quantum mechanical or enhanced sampling simulations [23,24].

For GH9, the picture is subtly different but no less interpretable. The protein scaffold remained compact and stable throughout (RMSD 2–5 Å; Rg 30.36 ± 0.33 Å), confirming a robustly folded enzyme. The mean hydrogen-bond occupancy of only 0.22—punctuated by transient peaks of up to five bonds—and the wide distribution of protein–ligand distances (2–40 Å, average 15.76 ± 11.19 Å) might superficially suggest weak binding, but this interpretation overlooks a key mechanistic feature of endoglucanases. These enzymes accommodate long, flexible polysaccharide chains within an open-channel active site and must allow the substrate to translocate by one or more sugar units after each catalytic cycle—a processive sliding mechanism rather than a lock-and-key interaction. The dynamic association and dissociation events sampled during the simulation are therefore consistent with, rather than contradictory to, productive cellulose hydrolysis. The elevated flexibility near residues 380–400 likely participates in this translocation by providing a conformationally adaptive surface that guides substrate repositioning. These computational observations connect directly to the strain’s experimentally characterized phenotype. The large endoglucanase clearance zone of 24.5 ± 1.2 mm on carboxymethyl-cellulose agar, the 11.6 ± 0.8 mm xylanase zone, and 63.4 ± 3.2% decolorization of alkali lignin are all consistent with the secretion of active GH9 and hemicellulose-targeting enzymes alongside oxidative CAZymes. The transient accumulation of ferulic acid (peak 0.85 mg/L on day 3) and vanillin (peak 0.67 mg/L on day 3), followed by depletion by day 6, reflects an orderly sequence of aromatic monomer release and subsequent catabolism that aligns with oxidative CAZyme activity—likely mediated by AA10 LPMO and AA3 oxidoreductase, both of which carried experimentally relevant cofactors (Cu^2+^ and FAD, respectively) in the homology models [23,24].

As summarized in (Table 4), the hydrogen yield of 1.41 mol H_2_ mol^−1^ substrate achieved by *B. subtilis* T7 from untreated food waste compares favorably with reported values for *Bacillus* strains operating on pure sugars or pretreated hydrolysates. While specific strains such as *B. coagulans* IIT-BT S1 have demonstrated higher yields (2.28 mol H_2_ mol^−1^ hexose) on glucose, these results typically depend on defined substrates and optimized batch conditions. Similarly, reports of elevated yields from *Bacillus* species often involve substrate pretreatment (e.g., acid/alkali hydrolysis of bagasse) or complex immobilized co-culture systems. In contrast, *B. subtilis* T7 operates within a simplified flowsheet: a single organism utilizing untreated waste without thermochemical pretreatment. Although the volumetric performance is slightly lower than the theoretical maximums observed with pure substrates, the elimination of pretreatment steps and the use of axenic culture on raw waste significantly reduce operational costs and energy input. This trade-off positions strain T7 as a viable candidate for consolidated bioprocessing applications where feedstock variability and pretreatment expenses are primary economic barriers.

Contextualizing these findings within the broader literature clarifies their significance. Previous studies have characterized hydrolytic and lignin-modifying activities in rumen-derived *Bacillus* strains; however, these works lacked docking or molecular dynamics data and relied on pretreatment steps alongside syntrophic consortia to achieve effective saccharification. Other research linked lignin peroxidase activity in tobacco-derived *B. subtilis* to redox gene upregulation via transcriptomics but stopped short of providing atomic-level binding insights. More recently, Yao et al. [25] characterized cellulase activity in *B. subtilis* Y4X3 isolated from saline–alkaline soil at the genomic level, offering a useful comparative framework for GH family conservation across *Bacillus* species. The T7 docking energies and protein stability metrics presented here exceed those reported for these systems and are among the most favorable documented for a naturally occurring *Bacillus* strain operating on cellulose and xylan substrates without genetic modification. This performance may reflect the selective pressure imposed by the anaerobic digester sludge environment from which T7 was isolated—a niche characterized by mixed and recalcitrant lignocellulosic inputs that would favor the evolution of tightly binding, catalytically versatile CAZymes.

It is important to acknowledge that hydrolytic competence and hydrogenogenic capacity are not always positively correlated within the *Bacillus* genus. Porwal et al. (2008) screened 35 bacterial isolates from diverse habitats and observed that strains exhibiting the highest lipase, protease, and amylase activities often differed from those with the highest hydrogen production potential [26]. In many cases, highly hydrolytic strains diverted significant carbon flux toward biomass and extracellular enzyme synthesis or toward reduced end-products like polyhydroxybutyrate (PHB), lactate, and ethanol, thereby limiting electron availability for the [FeFe]/[NiFe]-hydrogenase pool. The phenotype of *B. subtilis* T7—characterized by large hydrolytic clearance zones (cellulase: 24.5 mm; xylanase: 11.6 mm) coupled with a moderate hydrogen yield (1.41 mol H_2_ mol^−1^ substrate from untreated food waste)—aligns with this metabolic trade-off. While the molar yield is lower than values reported for *B. coagulans* or *B. thuringiensis* operating on pre-hydrolyzed sugars (Table 4), it reflects the energetic cost of simultaneous lignin modification and CAZyme secretion in a single organism. This balance allows T7 to function effectively as a standalone biocatalyst on untreated substrates, avoiding the complexity of multi-stage pretreatment or syntrophic consortia.

From a biotechnological perspective, the structural data carry concrete implications for enzyme engineering and bioprocess design. The key hydrogen-bonding residues identified in both Abhydrolase_1 and GH9 are primary targets for site-directed mutagenesis aimed at expanding substrate range, improving thermal stability, or enhancing catalytic rate. Overexpression of *hydA* (encoding [FeFe]-hydrogenase) combined with engineering of cofactor-regenerating pathways could further increase hydrogen flux, and CRISPR-based genome editing provides an efficient route to such modifications [27].

Because *B. subtilis* specifically is included in the GRAS list (not all *Bacillus* genus), any such engineered derivatives would face a more permissive regulatory pathway toward industrial deployment than would equivalent modifications in non-GRAS hosts. At the process level, a strain capable of directly valorizing the 1.3 billion tons of annual organic waste into hydrogen without separate pretreatment units, exogenous enzyme cocktails, or co-culture partners represents a meaningful simplification of the biorefinery flowsheet, with direct implications for capital cost and greenhouse gas footprint. The potential of *B. subtilis* T7 for such consolidated bioprocessing extends the current understanding of CBP beyond the predominantly anaerobic *Clostridium*-based systems, which typically require either substrate pretreatment or multi-organism consortia to achieve comparable conversion efficiency. Several limitations of the present study merit acknowledgment. Homology models, while constrained by high-quality templates and stringent quality filters (GMQE > 0.7; QMEAN > −4.0), may not capture all local structural nuances of the native enzyme—particularly in flexible loop regions that play key functional roles, as demonstrated by the RMSF data. The classical force field used in the MD simulations cannot describe bond-breaking events and therefore provides no direct information about catalytic mechanism or activation energy. Furthermore, both MD systems were equilibrated at physiological ionic strength in simple buffered water, whereas the actual enzyme environment during growth on food waste is far more complex. Future work integrating these computational models with quantitative proteomics and metabolic flux analysis of T7 grown on real lignocellulosic feedstocks will be essential to validate the predicted CAZyme activity patterns and guide rational strain improvement toward scalable, pretreatment-free biohydrogen production.

**Table 4:** Comparative hydrogen yields of *Bacillus* strains utilizing various substrates and fermentation systems. Values represent molar yield (mol H_2_ per mol hexose-equivalent).

Strain	Substrate	Mode	H_2_ Yield (mol H_2_/ mol Hexose)	Ref.
*Bacillus licheniformis*	Glucose/Biowaste	Free/Immobilized	0.58 (up to 2.34)	[28]
*Bacillus thuringiensis* EGU45	Sugarcane bagasse/Glycerol	Free/Immobilized	0.58–0.60	[29]
*Bacillus coagulans* IIT-BT S1	Glucose	Free cells, batch	2.28	[30]
*Bacillus coagulans* MO11	Molasses/Ethanol wastewater	Free cells	1.63	[30]
*Bacillus* spp. (Co-culture)	Cheese whey	Immobilized/Two-step	3.40 (Two-step) or 1.5 (Dark only)	[31]
*B. subtilis* T7	Untreated food waste	Free cells, axenic	1.41	This study

## 4. Materials and Methods

This study conducted an in silico structural characterization of carbohydrate-active enzymes (CAZymes) encoded in the genome of *B. subtilis* T7 to provide mechanistic support for previously reported experimental observations of lignin modification and biohydrogen production. The workflow proceeded in the following order: selection and homology modeling of target proteins with cofactor incorporation, preparation of ligands, blind molecular docking, site-specific docking, selection of the top-ranked complex for molecular dynamics simulation, execution and analysis of the simulation. All computations used publicly available tools and standard protocols to ensure reliability.

### 4.1. Ligand Preparation

Carbohydrate ligands were cellopentaose, xylotetraose, and cellobiose. Of these ligands, cellopentaose was used only for the inter-strain GH9 comparison reported in Supplementary Table S2; cellotetraose and xylotetraose were used for the 12-pair main-text docking screen, and the two highest-affinity complexes (Abhydrolase_1-xylotetraose and GH9-cellotetraose) were taken forward to molecular dynamics simulation and were prepared in three-dimensional form using the carbohydrate-specific GLYCAM06 force field through the GLYCAM-Web server (glycam.org). The structural sequences were either constructed manually or cross-checked using native oligosaccharides obtained from a homologous Protein Data Bank (PDB) structure (such as cellopentaose in PDB ID 7R2D), which ensured proper representation of the natural stereochemistry and β-(1→4) glycosidic bonds. The structures were initially minimized on the GLYCAM server to maintain the lowest-energy biologically active ^4^C_1_ chair conformation for the pyranose rings. The minimized coordinates were imported into AutoDockTools (V1.5.7) (ADT), where all polar hydrogens were added, non-polar hydrogens were merged, and Gasteiger partial charges were assigned. To preserve ring integrity during docking simulations, single bonds within the rings were set as non-rotatable (torsion tree audit). Conformational freedom of the polymer backbone was preserved while flexibility of the exocyclic groups and core glycosidic linkages (phi and psi dihedral angles) was systematically constrained, allowing realistic sampling of backbone geometry. Prior to conversion to AutoDock PDBQT format, all final configurations were validated by visual profiling and quantitative dihedral angle analysis in PyMOL (v2.5. Schrodinger, LLC) and compared against ideal dihedral ranges for the chair conformation.

### 4.2. Selection and Homology Modeling of CAZymes

Thirteen CAZymes implicated in lignocellulose degradation were selected from the annotated genome of *B. subtilis* T7. Three-dimensional structures were predicted using SWISS-MODEL (https://swissmodel.expasy.org). For each target, the template with the highest sequence identity, coverage, global model quality estimate (GMQE > 0.7), and QMEAN score (>−4.0) was chosen. Models were prepared for docking by adding polar hydrogens and assigning partial atomic charges in BIOVIA Discovery Studio 2021. Cofactor incorporation was performed for two oxidative enzymes. Cofactor placement was validated by conservation of coordinating residues and structural alignment with experimentally determined templates (RMSD < 1.0 Å for core regions). The highest-scoring pose that occupied the glycine-rich motif was retained.

### 4.3. Molecular Docking

Blind molecular docking was performed with AutoDock Vina (Version 1.2.7) to explore the global binding landscape and identify non-canonical and allosteric binding cavities on the target enzymes. Receptor proteins were prepared by removing water molecules, adding polar hydrogens, and assigning Kollman charges. A large grid box was centered on the geometric centroid of each protein structure with sufficient inter-grid-point spacing to encompass the entire solvent-exposed surface, thereby avoiding any a priori assumption about binding site location. The exhaustiveness parameter was set to a value of 8 or higher to enable thorough conformational sampling of the carbohydrate backbones across the full protein surface.

### 4.4. Site-Specific Molecular Docking

The binding affinity and precise molecular interactions of carbohydrate ligands with GH9 endoglucanase and Abhydrolase_1 were investigated through site-specific molecular docking. Binding cavities were predicted using the machine learning-based surface topography server PrankWeb. The center of mass of the top-ranked predicted pockets was used to define docking grid anchor points, and grid box dimensions were scaled dynamically to the physical volume and key residue composition of each pocket (20 Å to 30 Å). AutoDock Vina was used with a rigid receptor and flexible ligand. Final binding poses were ranked by binding free energy ΔG (kcal/mol) and evaluated for the orientation of the ligand within the catalytic clefts of each enzyme.

### 4.5. Molecular Dynamics Simulation

The selected enzyme–ligand complexes were subjected to all-atom molecular dynamics simulation using GROMACS 2023.2 and the CHARMM36 force field. Each system was solvated in a cubic box with TIP3P water molecules and neutralized with 0.15 M NaCl to approximate physiological ionic strength. Energy minimization was carried out with the steepest-descent algorithm until the maximum force fell below 1000 kJ mol^−1^ nm^−1^. Equilibration proceeded in two stages: 100 ps in the NVT ensemble and 500 ps in the NPT ensemble, both employing the V-rescale thermostat and Parrinello–Rahman barostat implemented in GROMACS 2020.6 (Manufacturer: GROMACS Development Team, Location: Groningen, The Netherlands), as coordinated by the Genomics & Informatics Lab (GIL), C-II, Johar Town, Lahore, Pakistan. Each production run lasted 100 ns with a 2 fs integration time step. Temperature was maintained at 300 K with the V-rescale thermostat, and pressure was held at 1 bar with the Parrinello–Rahman barostat. Covalent bonds involving hydrogen atoms were constrained with the LINCS algorithm. Three independent replicates were performed with randomized initial velocity seeds to assess reproducibility. Trajectory analysis employed standard GROMACS utilities; hydrogen-bond criteria were set at a donor–acceptor distance of 0.35 nm and angle of 30°.

### 4.6. Source and Characterisation of Food Waste, Fermentation Conditions, and Analytical Methods

Analytical method in this study focused on hydrogen gas, volatile fatty acid (VFA) accumulation, and lignin modification. While substrate conversion is implied by the stoichiometric yields and metabolite profiles, direct quantification of residual reducing sugars or volatile solids (VS) removal was not performed due to the analytical complexity of the untreated, heterogeneous food waste matrix.

Untreated food waste was collected from the central cafeteria at Shandong University of Technology (Zibo, China) and pooled over five consecutive days to ensure compositional homogeneity. The pooled material was homogenised in a high-speed blender to a particle size < 5 mm, divided into 200 g aliquots, and stored at −20 °C until use. No chemical (acid/alkali), enzymatic, or thermal pretreatment (e.g., autoclaving, steam explosion) was applied prior to fermentation. The biochemical composition of the dry matter was determined following standard Laboratory Analytical Procedures (LAP). The primary characterization study, the waste comprised 10.1 ± 0.3% glucan, 7.4 ± 0.2% xylan, 0.5 ± 0.1% arabinan, and 6.8 ± 0.2% lignin; mannan was not detected. The substrate contained 18.4 ± 0.4% total solids (TS) and 75.3 ± 1.1% volatile solids (VS) on a fresh weight basis, with a total organic carbon (TOC) content of 42.7 ± 0.9% of dry weight. Dark fermentation assays were conducted in 250 mL serum bottles with a 100 mL working volume. The basal medium contained (per L): NH_4_Cl 1.0 g, K_2_HPO_4_ 1.5 g, KH_2_PO_4_ 0.5 g, MgCl_2_·6H_2_O 0.2 g, FeCl_3_ 0.05 g, CaCl_2_ 0.05 g, NaHCO_3_ 4.0 g, and 1 mL trace element solution. Substrates (xylose, glucose, starch, CMC, or homogenised food waste) were added to a final concentration of 10 g/L (for pure substrates) or 12.5 g COD/L (for food waste). Bottles were inoculated with 5% (*v*/*v*) of a 24-h B. subtilis T7 pre-culture (OD_620_ ≈ 0.7), adjusted to pH 7.0 with 2 M NaOH, and flushed with N_2_ (>99.99%) for 5 min to establish anaerobic conditions before sealing with butyl rubber stoppers. Incubation proceeded at 37 ± 1 °C and 150 rpm for 96 h. Biogas volume was measured via water displacement, and gas composition (H_2_, CO_2_, CH_4_) was analysed by gas chromatography (GC) equipped with a thermal conductivity detector (TCD) and packed columns (Hayesep Q and Molecular Sieve 5A; N_2_ carrier). Hydrogen production kinetics were modelled using the modified Gompertz equation. Soluble sugars and volatile fatty acids (VFAs) were quantified by HPLC (Aminex HPX-87H column, 5 mM H_2_SO_4_ mobile phase, 0.6 mL/min, 65 °C, RI detection). COD was determined by closed-reflux dichromate digestion following Standard Methods (APHA). All experiments were performed in biological triplicate; data are presented as mean ± SD.

## 5. Conclusions

This in silico investigation provides molecular-level evidence supporting the lignocellulolytic potential of *Bacillus subtilis* T7. Structural modeling, blind docking, and site-specific molecular docking showed that GH9 endoglucanase and Abhydrolase_1 both possess accessible, energetically favorable binding regions for carbohydrate substrates. Among all enzyme–ligand combinations screened, Abhydrolase_1 showed the strongest affinity toward xylotetraose (−7.7 kcal/mol), consistent with a preference for hemicellulose-derived oligosaccharides, while GH9 preferentially recognized cellotetraose (−7.0 kcal/mol) and displayed the extended-groove geometry and aromatic stacking interactions characteristic of cellulolytic endoglucanases. Hydrogen bonding was the dominant stabilizing force in both complexes, with six and seven H-bonds identified for Abhydrolase_1 and GH9, respectively. One-hundred-nanosecond molecular dynamics simulations confirmed that both proteins maintained overall structural stability throughout the trajectory. The dynamic ligand behavior observed, partial xylotetraose displacement from Abhydrolase_1 after approximately 75 ns and intermittent association of cellotetraose within the GH9 catalytic channel, is consistent with the processive catalytic mechanisms of polysaccharide-degrading enzymes. The flexible loop region of Abhydrolase_1 (residues 170–180) and the mobile surface loop of GH9 (residues 380–400) stand out as candidate sites for future engineering efforts. These features help explain the strain’s experimentally observed endoglucanase and xylanase activities, 63.4% lignin modification, and direct biohydrogen production of up to 1.41 mol H_2_/mol substrate from untreated food waste. While limitations inherent to homology modeling and classical molecular dynamics apply, the results identify specific residues as engineering targets and validate *B. subtilis* T7 as a structurally and functionally coherent platform for sustainable, pretreatment-minimal consolidated bioprocessing.

## Figures and Tables

**Table 1:** Hydrogen yields and kinetic parameters from batch dark fermentation of various substrates by *B. subtilis* T7 (mean ± SD, n = 3).

Substrate	Cumulative H_2_ (mL/g VS)	Molar Yield (mol H_2_/mol Substrate)	R_max_ (mL/h)	Lag Phase (h)	Acetate (g/L)	Butyrate (g/L)	A:B Ratio (Molar)
Xylose	274 ± 15	0.92 ± 0.05	45.2 ± 3.1	12.4 ± 1.2	2.6 ± 0.3	1.6 ± 0.2	1.51
Glucose	193 ± 11	0.78 ± 0.04	38.6 ± 2.5	14.1 ± 1.5	2.2 ± 0.1	1.5 ± 0.3	1.36
Starch	132 ± 9	0.53 ± 0.03	35.4 ± 2.8	15.8 ± 1.3	1.3 ± 0.1	0.73 ± 0.3	1.66
CMC	169 ± 10	0.61 ± 0.04	32.1 ± 2.4	16.5 ± 1.6	1.4 ± 0.2	0.56 ± 0.1	2.32
Food Waste	246 ± 13	1.41 ± 0.08	40.8 ± 3.0	13.7 ± 1.4	1.7 ± 0.1	1.2 ± 0.2	1.31

Volatile fatty acids (VFAs) were quantified by HPLC (Aminex HPX-87H, 5 mM H_2_SO_4_, 0.6 mL/min, 65 °C, RI detection) at 96 h. The acetate-to-butyrate (A:B) ratio is expressed as a molar quotient. Propionate was undetectable (<0.05 g/L) in all runs. Kinetic parameters (R_max_: Represents the maximum hydrogen production rate, Lag Phase) were derived from the Modified Gompertz Model fit to cumulative hydrogen production data (R^2^ > 0.99 for all substrates).

**Table 2:** Best molecular docking scores of oligosaccharide ligands against carbohydrate-active enzymes.

Ligand	Target Protein	Binding Energy (kcal/mol)	Sequence Identity (%)	Reliable (≥30%)
Cellotetraose	AA3_1	−6.2	78.00	Yes
Xylotetraose	AA10_Cu_1	−5.6	100.00	Yes
Cellotetraose	Transporter_cytoplasmic	−6.1	91.94	Yes
Cellotetraose	Peptidase S8	−5.6	99.21	Yes
Xylotetraose	GH30	−6.1	91.94	Yes
Xylotetraose	GH11	−6.5	99.46	Yes
Cellotetraose	GH9	−7.0	100.00	Yes
Xylotetraose	Esterase	−5.9	94.30	Yes
Xylotetraose	Cellulase	−6.3	88.75	Yes
Xylotetraose	CE1	−5.9	100.00	Yes
Cellotetraose	Alpha-amylase	−6.4	98.63	Yes
Xylotetraose	Abhydrolase_1	−7.7	92.34	Yes

**Table 3:** Two highest interacting residues and hydrogen-bonding patterns identified from 2D ligand–protein interaction analysis from *B. subtilis* T7.

Ligand	Target	Binding Energy (kcal/mol)	Key Interacting Residues	No. of H-Bonds
Xylotetraose	Abhydrolase_1	−7.7	Gly32, Phe33, Thr34, Ser36, Ser39, His256, Ile180, Arg179	6 + 2 carbon Hydrogen
Cellotetraose	GH9	−7.0	Tyr141, Trp145, Asp194, Trp193, Arg254, Tyr255, Tyr354	7

## Data Availability

All the necessary data is included in the article. The genome was submitted to NCBI with accession number JBSSLY000000000 *B. subtilis* T7, submission ID SUB15686218, Biosample ID SAMN51985943 and Bioproject ID PRJNA1335374.

## Supplementary Materials

The following supporting information can be downloaded at: https://www.mdpi.com/article/10.3390/ijms27156610/s1.
