# Supplementary material for: In Silico Insights into Carbohydrate-Active Enzymes (CAZymes) of Bacillus subtilis T7: Lignin and Polysaccharide Degradation Mechanisms
Source: Int J Mol Sci. 2026 Jul 24;27(15):6610. doi: 10.3390/ijms27156610 (PMC13466493; doi:10.3390/ijms27156610)
Supplement: Supplementary file 1 [file ijms-27-06610-s001.zip › ijms-4408183-supplementary.pdf]

Supplementary data file

Table S1. Homology modelling templates, sequence identities, model-quality scores, and cofactors for the carbohydrate-active enzymes (CAZymes) of *Bacillus subtilis* T7 generated with SWISS-MODEL.

CAZyme Family	Enzyme	SWISS-MODEL Template	Seq. Identity (%)	GMQE	QMEAN	Cofactor
AA10	Lytic polysaccharide monooxygenase (LPMO)	6TWE	100.00	0.85	−1.20	Cu^{2+}
AA3	FAD-dependent oxidoreductase	6O9N	78.00	0.79	−2.10	FAD
GH9	Endoglucanase	8U4F (chain A)	100.00	1.01	−0.05	None
GH11	Endo-1,4-β-xylanase	2DCY (chain A)	99.46	0.86	0.94	None
GH30	Glucuronoxylanase	A0A7U0YJ67 (chain A)	91.94	0.94	−0.70	None
GH13 (α-amylase)	α-Amylase	P00691 (chain A)	98.63	0.91	−0.50	Ca^{2+}
CE1 (Abhydrolase_1)	Carbohydrate esterase	P70948 (chain A)	100.00	0.97	−0.60	None
Abhydrolase_1 (Pfam)	α/β-hydrolase	G4NZV8 (chain A)	92.34	0.97	−0.80	None
Peptidase S8	Subtilisin-like serine peptidase	P35835 (chain A)	99.21	0.91	−0.40	None

Note. CAZyme, carbohydrate-active enzyme; AA, auxiliary activity; GH, glycoside hydrolase; CE, carbohydrate esterase; LPMO, lytic polysaccharide monooxygenase; FAD, flavin adenine dinucleotide; GMQE, Global Model Quality Estimate (range 0–1; higher is better); QMEAN, Qualitative Model Energy Analysis (values close to 0 indicate native-like quality; values below −4.0 indicate low-quality models).

Templates are given as the SWISS-MODEL identifier (Protein Data Bank or UniProt accession with chain). Cu^{2+} and Ca^{2+} cofactors were incorporated into the AA10 and α-amylase models, respectively, based on conserved coordinating residues in the templates; FAD was modelled into the AA3 active site by structural superposition. All models met the inclusion thresholds GMQE > 0.70 and QMEAN > −4.0.

Table S2. Comparative molecular docking binding energies of cellopentaose (PubChem CID 16219172) against the GH9 endoglucanase of *Bacillus subtilis* T7 and homologous GH9 endoglucanases from related *Bacillus* species, ranked from strongest to weakest predicted affinity.

Rank	Source Organism (Strain)	Target GH9 Endoglucanase	Binding Energy (kcal mol^{-1})
1	*Bacillus subtilis* T7 (this study)	GH9 endoglucanase	−9.4
2	*Bacillus licheniformis*	Cel9Z	−8.8
3	*Bacillus pumilus*	BpGH9	−8.4
4	*Bacillus licheniformis* H1AD14	GH9 endoglucanase	−7.9
5	*Bacillus subtilis* K35-1	GH9 endoglucanase	−7.2
6	*Bacillus velezensis*	GH9 endoglucanase	−7.0
7	*Bacillus subtilis* IARI-SP-1	GH9 endoglucanase	−6.3

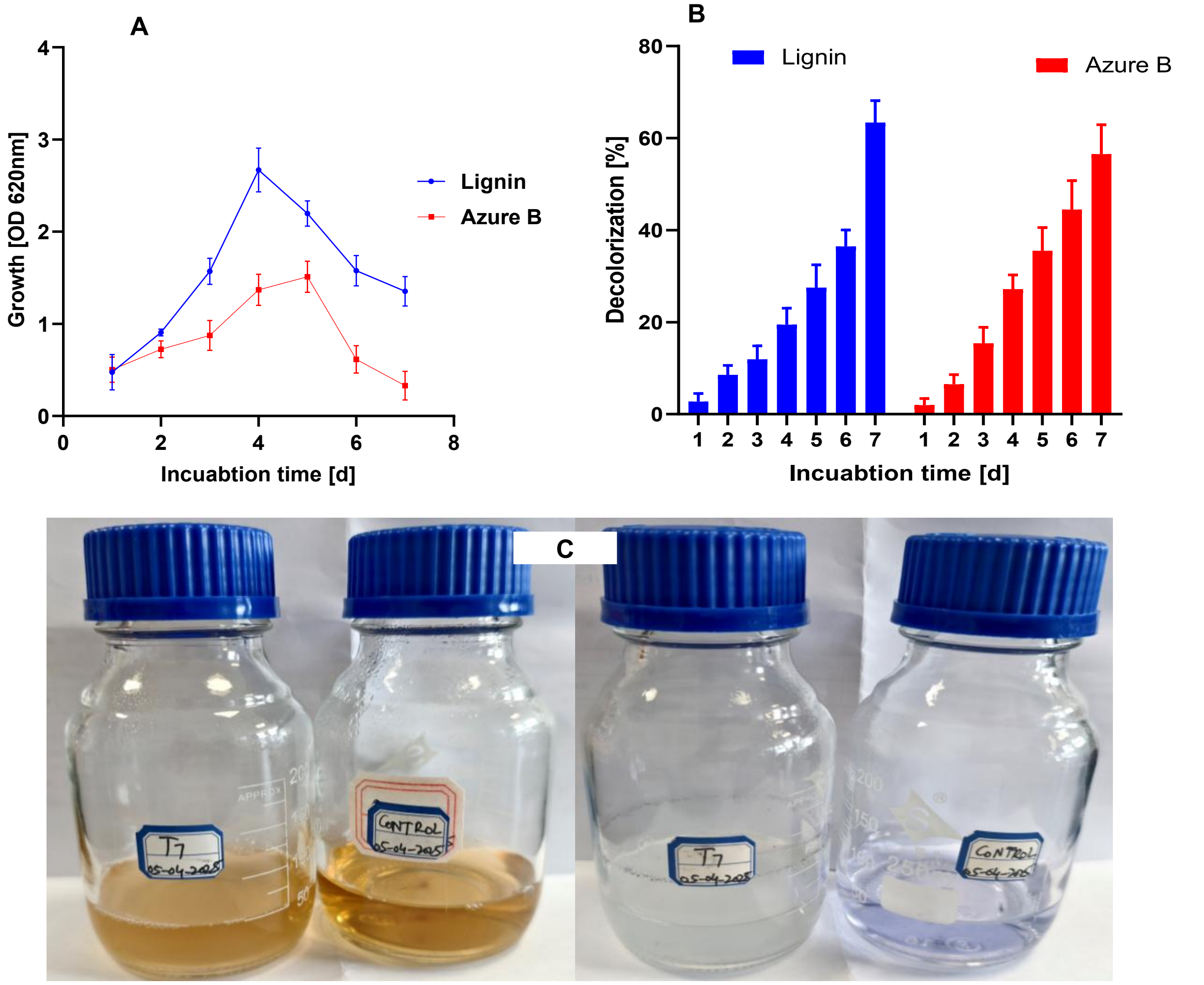

Lignin decolorization of T7

Azure B decolorization of T7

Supplementary Figure. S1. (a) *B. subtilis* T7 growth (OD_{620}) on lignin- or Azure B-supplemented MSM over 7 days at 37 ± 1°C, 150 rpm. (b) Decolorization (%) of lignin (280 nm) and Azure B (663 nm) by T7 over 7 days, visual decolorization of lignin and Azure B in MSM by T7 over 7 days (Fig. 1c).

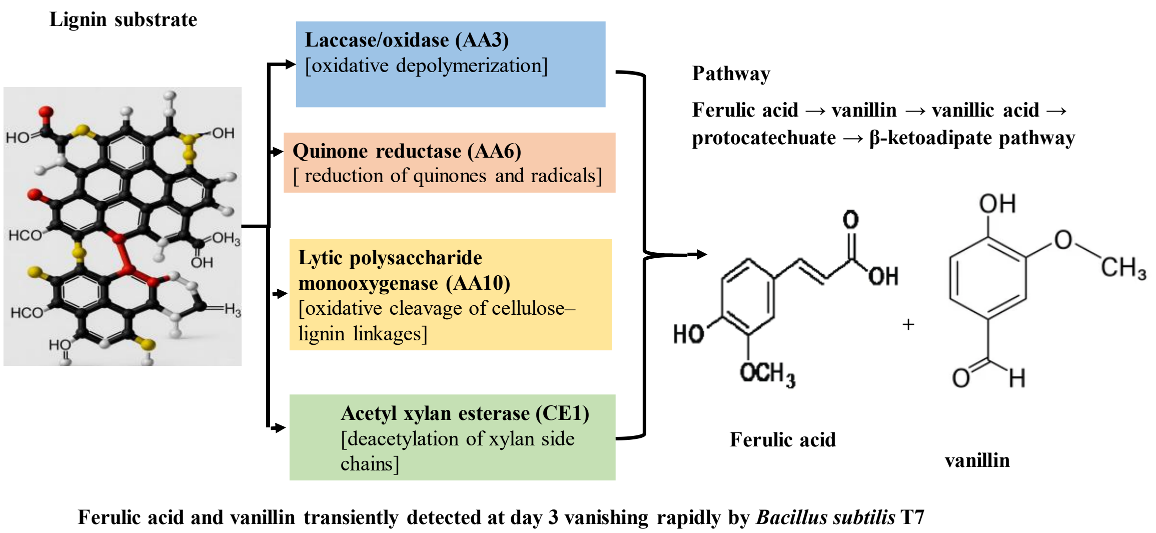

Supplementary Figure S 2: Mechanism of lignin conversion into ferulic acid and vanillin and key genes involved in *Bacillus Subtilis* T7

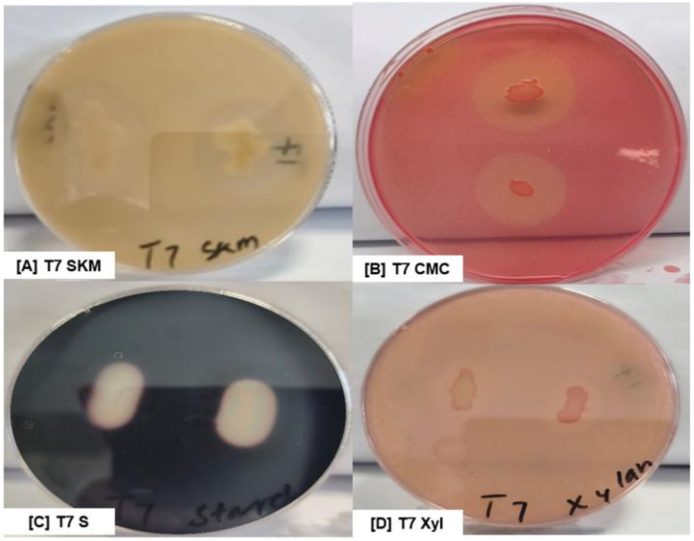

Supplementary Figure S3: Hydrolysis zones by T7 on skim milk (proteases), CMC (cellulases), starch (amylases), and xylan (xylanases) agar plates

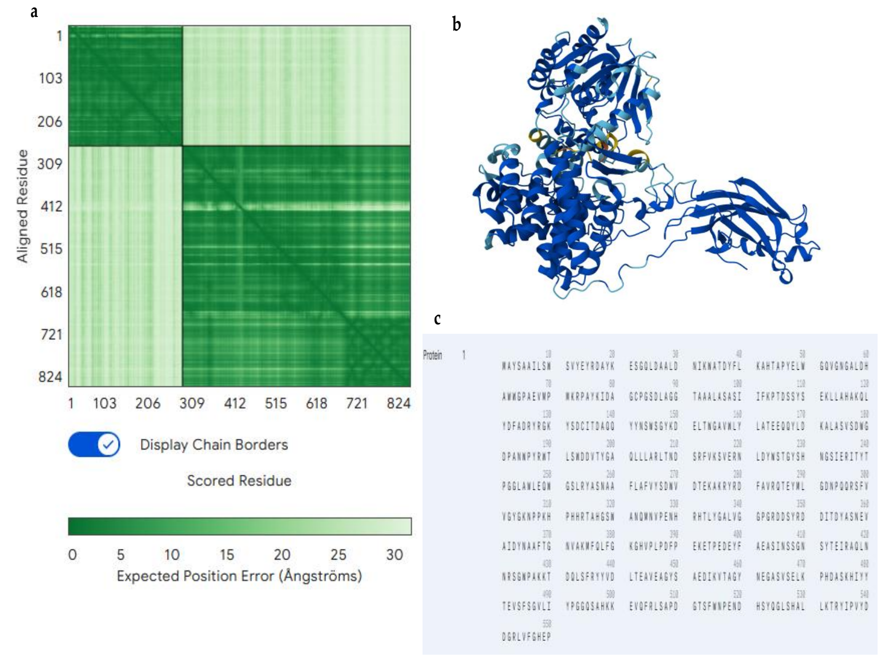

Supplementary Figure S4. AlphaFold prediction of the GH9 . (a) *Predicted Aligned Error (PAE) plot showing the confidence in the relative positions of residue pairs.* ***(b)*** *Three-dimensional structure predicted by AlphaFold, colored according to pLDDT confidence scores (dark blue, very high confidence; light blue, confident; yellow, low; orange, very low).* ***(c)*** *Model confidence metrics, including* ***pTM = 0.67*** *and* ***ipTM = 0.18 and protein FASTA sequence***

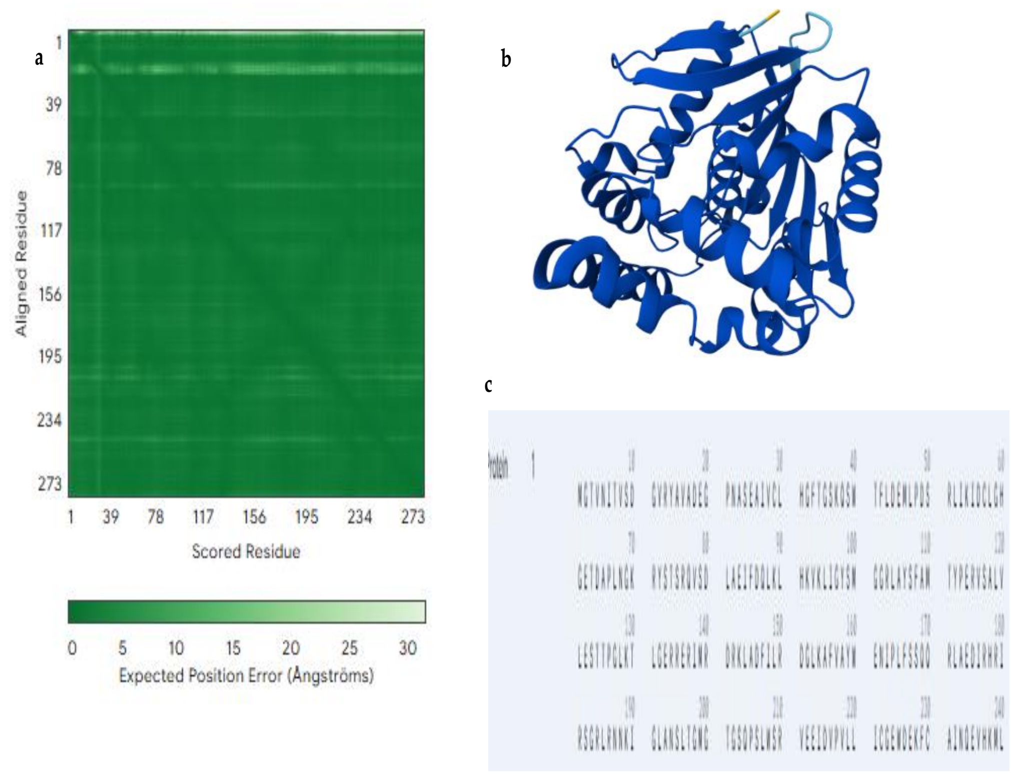

Supplementary Figure S5. AlphaFold prediction of the Abhydrolase-1. (a) *Three-dimensional structure predicted by AlphaFold and colored according to pLDDT confidence scores.* ***(b)*** *Predicted Aligned Error (PAE) plot showing confidence in residue-pair positioning.* ***(c)*** *Model confidence metrics with* ***pTM = 0.96 and protein FASTA sequence****.*
